# Listeriosis: A Rare Cause of Cardiac Implantable Electronic Device Infection

**DOI:** 10.7759/cureus.72870

**Published:** 2024-11-02

**Authors:** Fábio Pé D’Arca Barbosa, Gonçalo Cristóvão, Maria Carlos, Diana Seixas, Fernando Maltez

**Affiliations:** 1 Internal Medicine, Hospital Garcia de Orta - Unidade Local de Saúde Almada-Seixal, Almada, PRT; 2 Infectious Diseases, Hospital Curry Cabral - Unidade Local de Saúde São José, Lisbon, PRT

**Keywords:** cardiac implantable device management, endocarditis, gastroenteritis complication, invasive listeriosis, pacemaker infection

## Abstract

Listeriosis is a series of diseases caused by the bacteria *Listeria monocytogenes*. Typically presenting with mild symptoms of diarrhea, fever, and myalgias, it can, in some cases, assume an invasive form with systemic involvement. Cardiac involvement in listeriosis is rare, with very few reports describing the involvement of cardiac implantable electronic devices (CIED), which hampers the development of conclusive recommendations regarding CIED management or antibiotic courses.

We present the case of an 82-year-old woman who had a pacemaker and developed gastroenteritis complicated by bacteremia due to *L. monocytogenesis*. The presence of vegetation in one of the pacemaker leads led to the diagnosis of endocarditis and subsequent lead substitution along with effective antibiotic therapy. This case highlights the importance of thorough investigation in patients with bacteremia due to non-typical microorganisms for infective endocarditis and describes a possible approach for device removal.

## Introduction

Listeriosis is an infection caused by *Listeria monocytogenes*, a gram-positive, facultative, intracellular bacterium that primarily affects individuals with weakened immune systems [1]. It can cause a broad spectrum of clinical presentations with two major forms of the disease having been described: a non-invasive form known as febrile listerial gastroenteritis, presenting with mild self-limiting gastroenteritis, and an invasive form, which often involves dissemination and systemic infection such as sepsis or meningitis [1].

Cardiac involvement in listeriosis is exceedingly rare, yet potentially life-threatening. It has been reported to affect up to 8% of patients, with only 101 cases reported from 1955 until 2018, associated with a mortality rate of 37-50% [2]. The most commonly affected structures in cardiac listeriosis are the aortic and mitral valves [2]. Cardiac implantable electronic devices (CIED)-related infections are typically associated with more common pathogens such as Staphylococcus aureus or coagulase-negative staphylococci. The presence of *Listeria* in CIED infections represents a unique clinical challenge due to the scarcity of clinical data, making it difficult to ascertain an optimal therapeutic strategy. Current evidence regarding the management of CIED infections is largely based on cases involving more common pathogens, and the role of specific antibiotic regimens and device management in the setting of *Listeria* infection remains unclear [3-6].

In this article, we present a rare case of *Listeria monocytogenes *endocarditis with CIED involvement. We aim to discuss its clinical presentation, management, and the considerations around the timing of CIED extraction and re-implantation, as well as the implications of effective antibiotic therapy, aiming to contribute to the growing body of evidence regarding the approach to CIED management in these cases.

## Case presentation

We present the case of an 82-year-old woman with a known past medical history of heart failure with a reduced ejection fraction due to both ischemic and valvular heart disease, third-degree atrioventricular heart block with the implantation of a pacemaker three years prior to current admission, hypertension, localized colon cancer treated with right hemicolectomy three years prior to current admission without evidence of recurrence or relapse, and chronic kidney disease (CKD), staging 3a according to glomerular filtration rate and the Kidney Disease Improving Global Outcomes (KDIGO) classification.

**Figure 1:**
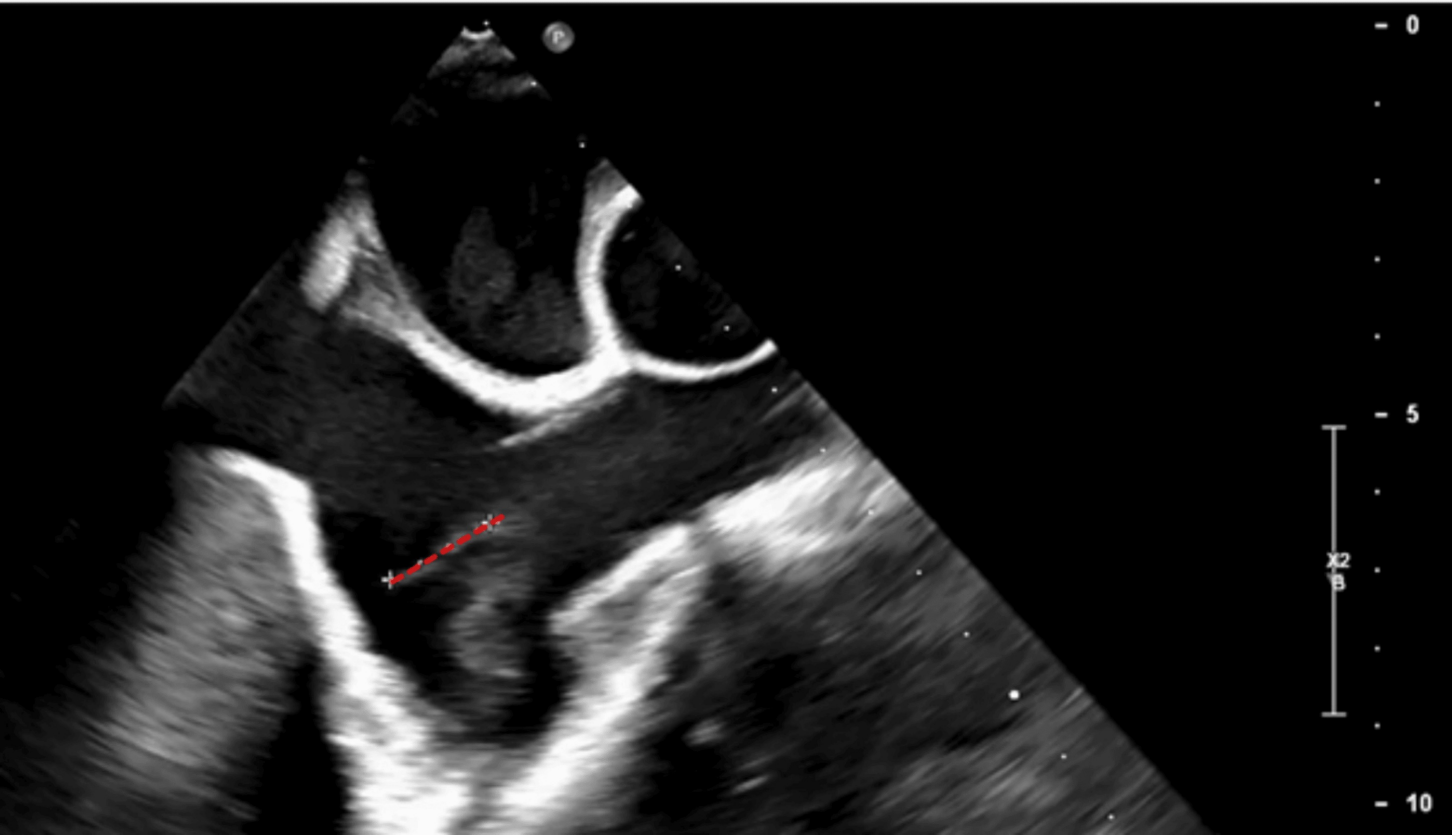
Mid-position transoesophageal echocardiography image A mass measuring approximately 15 millimeters can be seen attached to one of the pacemaker leads in the right atrium (red dotted line in the image, representing the measurement).

The patient presented to the emergency department with a two-day history of fever, diffuse colicky abdominal pain, and watery diarrhea without blood or mucous. On admission, she was hypotensive, febrile, and had abdominal pain in all quadrants on abdominal palpation. Her blood work revealed leucocytosis, an elevated C-reactive protein level, and acute renal injury (Acute Kidney Injury Network stage 2). Blood and stool samples were collected for culture in search of the culprit pathogen. The patient was admitted as an inpatient and empirically started on antibiotics (ceftriaxone) and fluid therapy. She was discharged home with oral amoxicillin/clavulanate after three days of inpatient treatment and after having resolved the renal injury. During a follow-up appointment four days after discharge, it was noticed that the blood cultures had tested positive for *L. monocytogenes*. She was readmitted for intravenous treatment with ampicillin as per pathogen susceptibility. Follow-up blood cultures were negative, and throughout admission, the patient remained afebrile and hemodynamically stable. A transthoracic echocardiogram showed no signs of endocarditis albeit conditioned by a weak acoustic window. Subsequently, she underwent a transoesophageal echocardiogram, which revealed the presence of a 15-millimeter vegetation on one of the pacemaker leads (Figure 1, Video 1).

**Video 1:** Mid-position transoesophageal echocardiography video A movie clip showing a mobile mass measuring approximately 15 mm can be seen, attached to one of the pacemaker leads in the right atrium.

She completed a four-week course of intravenous ampicillin before undergoing pacemaker lead removal via a percutaneous approach. Re-implantation was performed after five days using the same route. The patient received an additional two weeks of intravenous ampicillin after lead removal. During the follow-up period of five months, the patient did not present with signs of recurrent infection.

## Discussion

Accurately estimating the global incidence of *Listeria* infections is difficult, and the true prevalence may be higher than previously assumed. Recent evidence suggests that these infections are increasingly linked to more severe clinical outcomes [7].

We present a clinical case of invasive listeriosis involving a CIED, highlighting the complexities of its diagnosis and management. Initially manifesting as febrile listerial gastroenteritis, this patient had risk factors for invasive disease such as the presence of an implantable cardiac device, CKD, and known valvular disease [8]. In our patient, transoesophageal echocardiogram (TEE) revealed a significant lead vegetation despite an initially negative transthoracic echocardiogram (TTE), highlighting the crucial role of TEE, especially in cases where TTE may not provide sufficient detail due to poor acoustic windows or small vegetations.

Current guidelines for the management of CIED infections recommend prompt removal of the device within the first 72 hours of diagnosis [4,5]. However, in cases of* Listeria* endocarditis, the scarcity of clinical data has led to variations in management strategies. In our patient, we opted for a delayed approach to device extraction, continuing antibiotic therapy for two weeks before proceeding with pacemaker lead removal. This decision was based on the rationale that reducing the size of the vegetation prior to extraction could potentially decrease the risk of septic embolism, a known complication during lead removal procedures [4,5,9]. The safety and efficacy of this approach have been demonstrated in similar cases, where prolonged antibiotic therapy was employed either prior to surgical intervention [8] or as the only means for treatment should the patient be unfit for interventional strategies [10,11].

Although no randomized controlled trials exist to determine the ideal timing for the re-implantation of CIEDs following removal, most experts suggest delaying re-implantation to minimize the risk of reinfection [5]. In our patient, the pacemaker was re-implanted five days after lead extraction, following five days of directed antibiotic therapy. This timeline allowed for sufficient time to ensure clinical stability and prevent the recurrence of infection. Post-extraction antibiotic therapy was continued for an additional two weeks, which is consistent with the recommendations for treating infective endocarditis without valvular involvement [4,5].

The favorable outcome in our patient, who remained free of infection recurrence and CIED malfunction during a five-month follow-up, suggests that a patient-tailored approach to both the timing of device extraction and re-implantation, combined with adequate antibiotic coverage, can be safe and effective in managing such cases. Further clinical studies are needed to define standardized protocols for the management of *Listeria monocytogenes* infections involving CIEDs, as well as to determine the optimal timing for device re-implantation.

## Conclusions

A *Listeria Monocytogenes *infection should be raised as a possibility in a patient presenting with febrile gastroenteritis. Cardiac involvement is rare and can lead to death. In patients with CIEDs, the optimal approach to treatment is not well-established. With this case, we try to demonstrate the safety and efficacy of a delayed approach for CIED removal, allowing for a reduction in vegetation size and lower embolic risk during lead extraction, adding to current evidence and highlighting the importance of patient-tailored approaches.
